# Multimerization of a rationally designed nanobody for enhanced avidity toward Aβ42 oligomers

**DOI:** 10.1002/pro.70754

**Published:** 2026-08-19

**Authors:** Magdalena Nowinska, Elijah Suh, Casey Mogilevsky, Michele Vendruscolo

**Affiliations:** ^1^ Centre for Misfolding Diseases, Yusuf Hamied Department of Chemistry University of Cambridge Cambridge UK

**Keywords:** Alzheimer’s disease, avidity, Aβ42 oligomers, multivalency, nanobodies, protein aggregation

## Abstract

Alzheimer's disease affects tens of millions of people worldwide and is associated with the self‐assembly of the Aβ42 peptide into amyloid aggregates. Among the species formed during this process, soluble oligomeric intermediates are the most closely linked to neurotoxicity and are therefore an attractive target for both therapeutic and diagnostic strategies. Their conformational heterogeneity and transient nature, however, have so far hindered the development of reagents that recognize them selectively, and no fully quantitative biomarker of Aβ42 oligomers is widely available. To address this problem, we use a rationally designed conformation‐specific single‐domain antibody, DesAbO, which binds selectively to Aβ42 oligomers. By using enzyme‐linked immunosorbent assay, we show that encoding self‐assembling multimerization domains in the DesAbO plasmid yields multimeric variants with increased avidity toward Aβ42 oligomers. In aggregation assays, the multimeric variants inhibited Aβ42 aggregation at concentrations at which the monomeric form was no longer effective, with the SB175 trimer performing best. These results show how multimerization can be used to enhance the recognition of Aβ42 oligomers and offer a route toward diagnostic and therapeutic agents for Alzheimer's disease and other protein misfolding disorders.

## 1 INTRODUCTION

Alzheimer's disease is the most common cause of dementia, with projections indicating that it will afflict over 150 million patients worldwide by 2050 (Knopman et al., 2021; Scheltens et al., 2021) unless effective treatments become widely available (Cummings, Osse, et al., 2024; Cummings, Zhou, et al., 2024; Long & Holtzman, 2019).

A molecular hallmark of Alzheimer's disease is the accumulation of insoluble protein deposits, most notably amyloid plaques and neurofibrillary tangles, in specific brain tissues (Hampel et al., 2021; Hardy & Selkoe, 2002; Jack Jr et al., 2018). Amyloid plaques, in particular, predominantly consist of aggregated forms of the amyloid β peptide (Aβ) (Hampel et al., 2021; Hardy & Selkoe, 2002; Jack Jr et al., 2018) and are formed through a complex process that involves a variety of structurally different species (Cohen et al., 2013; Michaels et al., 2020). Among such species, increasing evidence has implicated small, soluble oligomers as major agents responsible for neurotoxicity in Alzheimer’s disease (Benilova et al., 2012; Haass & Selkoe, 2007). In particular, these oligomers have been shown to trigger various toxic pathways, including synaptic dysregulation, membrane permeabilization, oxidative stress, mitochondrial dysfunction, and activation of pro‐inflammatory response (Benilova et al., 2012; Haass & Selkoe, 2007; Rinauro et al., 2024; Shankar et al., 2008). Recent anti‐Aβ antibodies, including aducanumab (Sevigny et al., 2016), lecanemab (Johannesson et al., 2024; Van Dyck et al., 2023), and donanemab (Mintun et al., 2021; Sims et al., 2023), reduce brain amyloid burden and modestly slow clinical decline in early Alzheimer's disease, although they differ in their recognition of soluble and fibrillar Aβ assemblies (Linse et al., 2020).

The conformational heterogeneity, low concentrations, and transient nature of these oligomeric species have made their isolation and characterization very challenging. Antibodies offer a variety of opportunities to overcome this challenge, as they represent powerful and versatile tools, owing to their high binding specificity and affinity and well‐established discovery methods (Bradbury et al., 2011; Winter et al., 1994). These protein molecules have highly successful applications in diagnostics, therapeutics, and targeted drug delivery systems for infectious diseases, cancer, and metabolic and hormonal disorders (Leader et al., 2008). In particular, many diagnostic tests routinely used in the clinic are based on antibodies. For this reason, in the last 20 years, major efforts have been made to overcome the challenges in isolating and stabilizing oligomeric species for immunization and phage display protocols to develop antibodies that selectively recognize such species in positron emission tomography scans and biological samples from patients (Kayed et al., 2003; Kulenkampff et al., 2021; Sehlin et al., 2016; Yang et al., 2019).

The large size of conventional monoclonal antibodies (~150 kDa), however, has hampered in vivo efforts to target tissues and cross the blood–brain barrier. Single‐domain antibodies, which can be as small as 15 kDa, have therefore been developed (Jovčevska & Muyldermans, 2020). Also known as nanobodies (Nbs), these naturally occurring, single‐V‐domain structures (called variable domain of the heavy chain of a heavy‐chain‐only antibody domains) retain the affinity of larger antibody molecules while being of smaller size. With this technology, a rationally designed conformation‐specific Nb, DesAbO, was shown to selectively bind to oligomers of Aβ42 (the 42‐residue form of Aβ), rather than to its monomeric or fibrillar forms (Aprile et al., 2020; Bigi et al., 2024). More recently, a tandem dimer of DesAbO, termed DiDesAbO, was reported to enhance the detection and neutralization of Aβ42 oligomers relative to monomeric DesAbO, including in neuronal‐cell assays and cerebrospinal‐fluid samples from patients with Alzheimer's disease (Napolitano et al., 2026).

In this work, we extend this multimerization strategy by systematically comparing self‐assembling DesAbO architectures ranging from trimers to octamers, with the aim of determining how multimer geometry and valency influence binding to Aβ42 oligomers. Multimeric Nbs that specifically target Aβ42 oligomers could be used in two main ways. First, as biomarkers in diagnostic assays, as the concentration of soluble oligomers in the cerebrospinal fluid is estimated to be in the pM range or lower (Georganopoulou et al., 2005; Kulenkampff et al., 2021). Second, as therapeutics, these constructs hold promise for delivering precise and effective treatment strategies.

Overall, we aimed to enhance the utility of the DesAbO Nb by engineering multimeric variants with increased avidity and specificity for Aβ42 oligomers. By designing and evaluating different multimer types, including trimers (SB175 and T4 Fibritin), tetramers, pentamers, hexamers, and octamers, we systematically assess their binding properties and aggregation‐inhibition capabilities. These constructs use diverse multimerization domains to explore the relationship between multimer architecture and functional performance.

## 2 RESULTS AND DISCUSSION

### 2.1 Design of DesAbO multimeric variants

**FIGURE 1:**
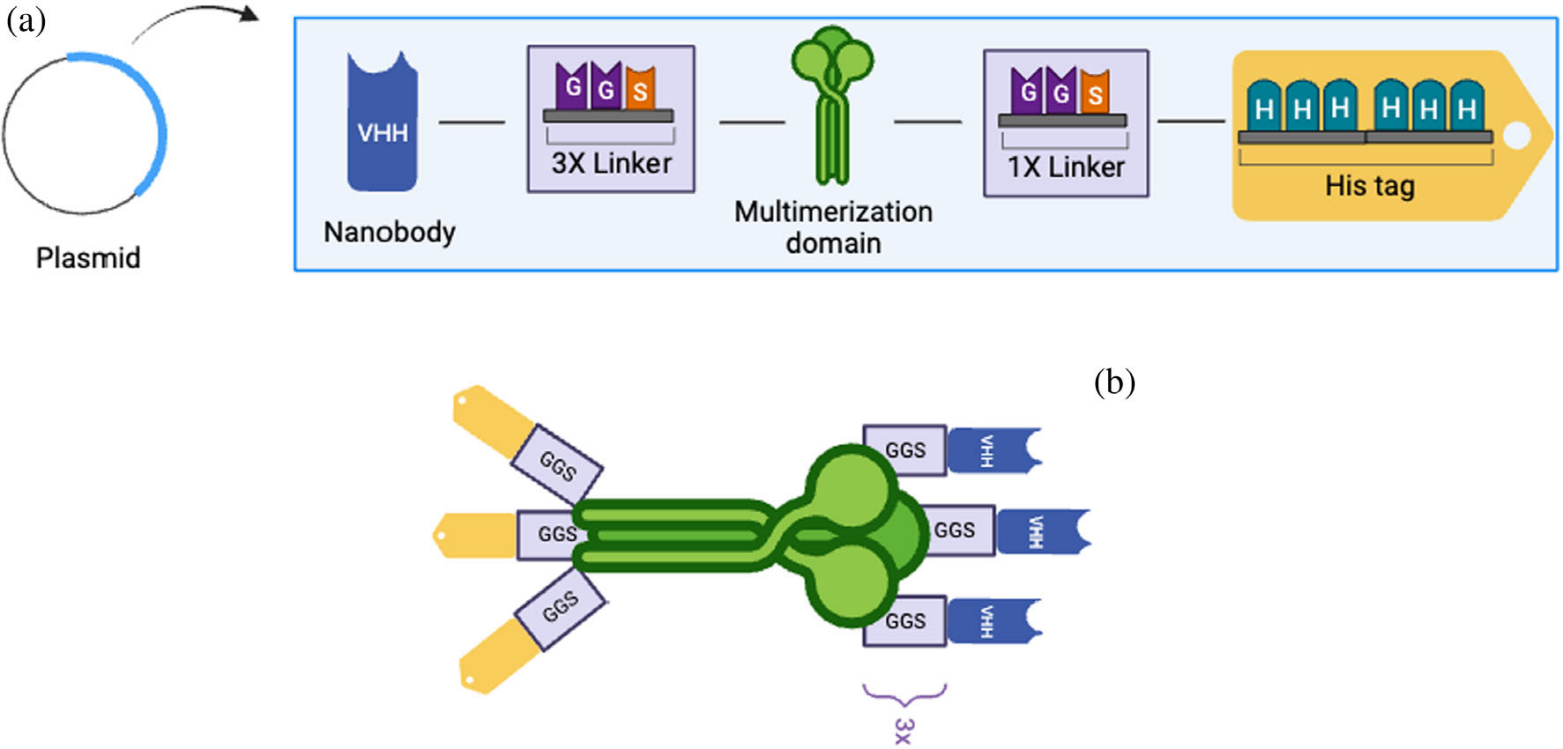
Schematic representation of the procedure used in this work for nanobody multimerization. (a) Components encoded in the plasmid required for multimerization of the nanobody (VHH, blue). The multimerization domain (depicted as a trimer, green) functions as the scaffold that holds the nanobodies together. The multimerization domain is connected to the nanobody via a (GGS)_3_ linker and to a 6× His tag via a (GGS)_1_ linker (purple). The His tag (yellow) is used during protein purification and the enzyme‐linked immunosorbent assay measurements. (b) Example, in the case of a trimeric variant, of the result of the self‐assembly process.

Multimerizing DesAbO increased its avidity. Multivalent and multimeric antibodies have been engineered using three main strategies: (i) linking monovalent Nbs to protein cages, such as the *Aquifex aeolicus* lumazine synthase (AaLS) (Lu et al., 2023); (ii) assembling Quad molecules through p53 tetramerization domains (Wade et al., 2022), and (iii) employing the SpyTag/SpyCatcher superglue system to enhance antibodies against Severe Acute Respiratory Syndrome Coronavirus 2 (Sun et al., 2025). In particular, a homotrimeric minibinder fusion produced multivalent miniprotein agents (Hunt et al., 2022). More directly related to the present work, two DesAbO units connected by a flexible peptide linker were recently shown to exhibit enhanced Aβ42 oligomer binding and anti‐aggregation activity compared with monomeric DesAbO (Napolitano et al., 2026). Here, rather than covalently linking DesAbO units in tandem, we use self‐assembling multimerization domains to generate and compare architectures of different valencies and geometries. The general scheme to combine DesAbO, including oligomerization domains and linkers, is shown in Figure 1.

### 2.2 Binding assessment

**FIGURE 2:**
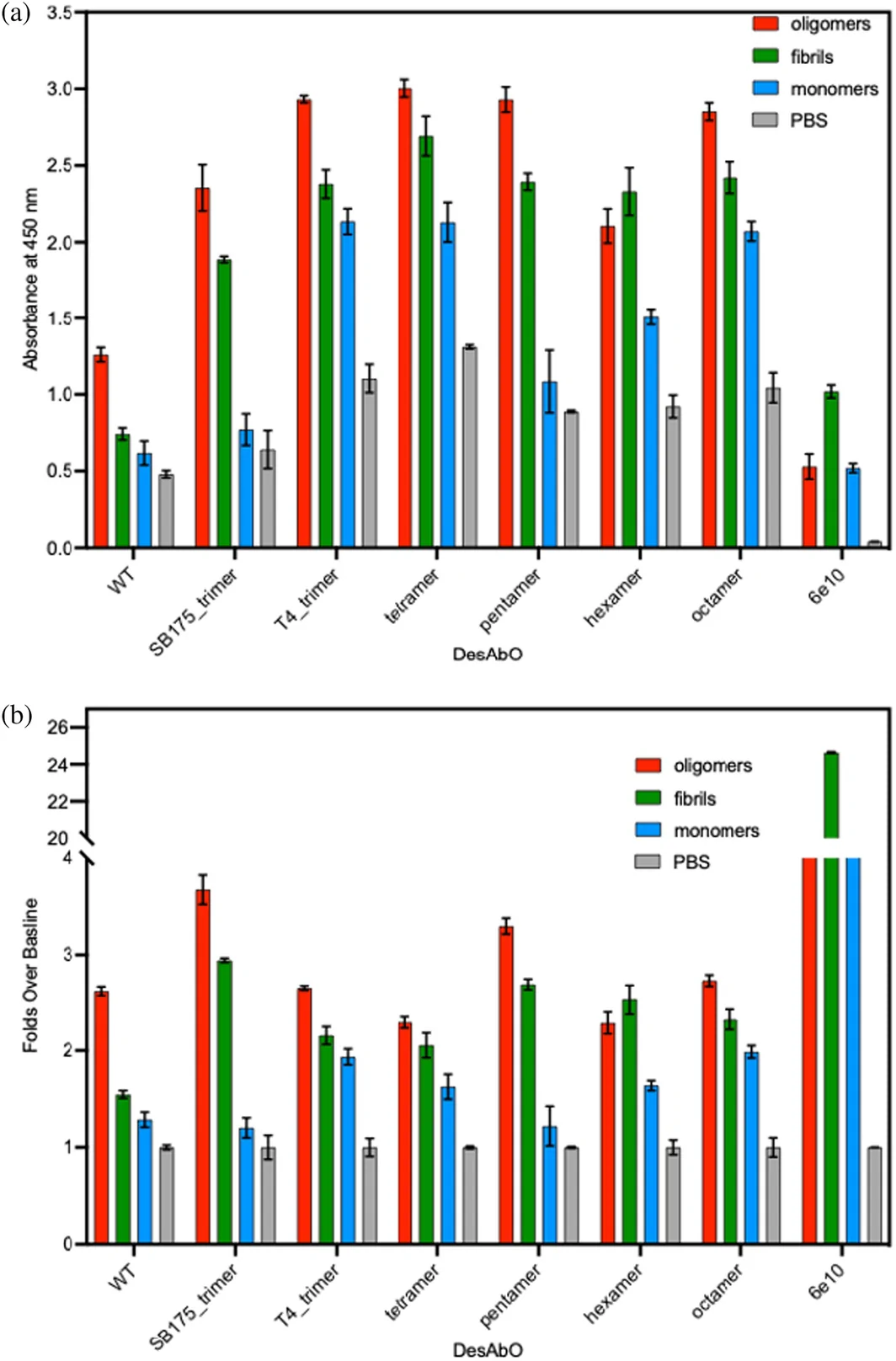
Analysis of the binding of DesAbO multimers to Aβ42 monomers, oligomers, and fibrils with an indirect enzyme‐linked immunosorbent assay (ELISA). (a) The DesAbO multimers exhibited higher binding signals to Αβ42 oligomers (red) compared to monomers (blue) and fibrils (green), with each multimeric variant having higher binding signal compared to DesAbO (wild‐type [WT]). Phosphate‐buffered saline (PBS) (gray) was used as negative control. The binding was assessed by the absorbance at 450 nm. (b) ELISA results plotted as folds‐over‐baseline. Data were normalized to the lowest nanobody‐antigen measurement of each multimeric variant (PBS). In both panels, error bars represent the standard deviation.

Following the purification and characterization of DesAbO and its multimeric variants (see Section 4), we measured the binding of the DesAbO multimers to Αβ42 oligomers, fibrils, and monomers. To assess the ability of DesAbO multimers to detect Aβ42 oligomers, we compared their performance to that of 6E10, a commercial antibody that targets the N‐terminus of Aβ42 and binds both Aβ42 monomers and Aβ42 aggregates. To assess the binding, we employed an indirect enzyme‐linked immunosorbent assay (ELISA), where we immobilized Aβ42 on the plate and used DesAbO and its multimeric variants as detection antibodies (Figure 2a). We found that the binding signals of the DesAbO multimers were increased relative to DesAbO. We also observed that DesAbO and its multimeric variants were more selective toward Aβ42 oligomers over monomers and fibrils than the commercial antibody 6E10. To better evaluate the relative binding of the DesAbO multimeric variants toward the three Aβ42 species, we plotted the ELISA data as folds‐over‐baseline (FOB) values (Figure 2b). This normalization helps account for plate‐to‐plate variability, Nb‐concentration differences, and background signal. This is particularly useful in assessing the relative potency of the multimeric variants, as the increase in valency generally increased binding for all antigens, not just Αβ42 oligomers.

The SB175 trimer, the pentamer, and the octamer showed the highest FOB values for Aβ42 oligomers (Figure 2b), indicating that these architectures retain conformational preference for oligomers over monomers and fibrils. The tetramer behaved differently, as it gave the highest absolute binding signal toward Aβ42 oligomers of any construct (Figure 2a), but its signals toward monomers and fibrils increased in parallel, so that its FOB value was lower (Figure 2b). Valency alone, therefore, does not determine performance, since multimerization increases the signal toward all three Aβ42 species and only some geometries increase it preferentially for oligomers. The hexamer, the construct of highest valency after the octamer, gave both the lowest absolute signal and among the lowest FOB values of the multimers (Figure 2a,b), which suggests that steric constraints or unfavorable epitope–paratope orientation can offset the gain in avidity at higher valency.

The ELISA measurements reported here should be interpreted as comparative binding signals rather than as absolute affinities or kinetic constants. Quantitative determination of multivalent binding parameters against Aβ42 oligomers is not straightforward, since the oligomers are heterogeneous in size, transient and themselves multivalent, so that any measured constant convolves intrinsic affinity, avidity, the oligomer size distribution, and the binding stoichiometry. Obtaining interpretable values by surface plasmon resonance, biolayer interferometry, or isothermal titration calorimetry would require oligomer populations of defined and independently characterized size distribution, together with an analysis able to separate these contributions, and represents an important direction for future work.

In the structural models (Figure 6), the paratopes of the SB175 trimer are more closely spaced (~36 Å) than those of the tetramer (~44 Å). A spacing better matched to the separation of accessible epitopes on a single oligomer would favor simultaneous engagement and hence selectivity, whereas a wider spacing would be less able to achieve this while remaining compatible with the closely and repetitively spaced epitopes of a fibril surface. Because the epitope spacing on Aβ42 oligomers is not known, and because the models do not capture linker flexibility, these distances should be read as a geometric hypothesis rather than as a demonstrated epitope–paratope match. We have not measured the binding stoichiometry, and the indirect ELISA format used here cannot distinguish multivalent engagement within a single immobilized assembly from bridging or rebinding between neighboring ones.

### 2.3 Non‐specific binding analysis

**FIGURE 3:**
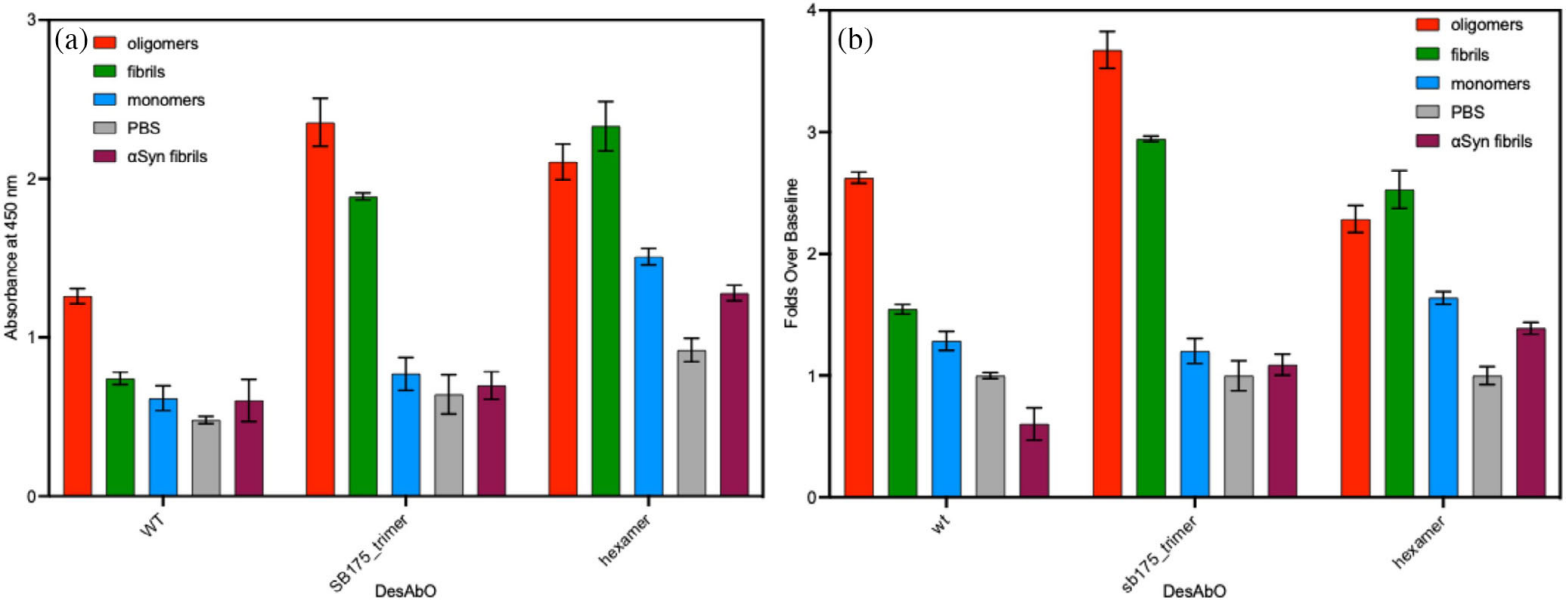
DesAbO multimers preferentially bind Aβ42 oligomers over α‐synuclein fibrils. (a) Comparison of the binding signal of two DesAbO multimeric variants (a trimer and a hexamer) with α‐synuclein fibrils (purple), and Αβ42 oligomers (red), fibrils (green) and monomers (blue), and phosphate‐buffered saline (PBS) as negative control (gray). (b) Enzyme‐linked immunosorbent assay folds‐over‐baseline normalization graph. Data were normalized to the lowest nanobody‐antigen measurement of each multimeric variant (PBS). In both panels, error bars represent the standard deviation.

To assess the binding specificity of DesAbO multimers for Αβ42, we tested whether they could bind α‐synuclein, an amyloidogenic protein involved in Parkinson's disease. α‐Synuclein, similar to Αβ42, also forms oligomers and fibrils rich in β‐sheet structures. Previous examinations underscored the conformational and sequence selectivity of DesAbO, as shown by its minimal binding to α‐synuclein oligomers (Aprile et al., 2020). We investigated the hexameric variant, the worst‐performing multimeric variant, and the SB175 trimeric variant, one of the most promising DesAbO multimers (Figure 3a). We found that DesAbO multimers had a significant preference for Aβ42 oligomers over α‐synuclein fibrils (Figure 3a). The binding to α‐synuclein was comparable to the background noise. As above, we also normalized the data, presenting it as FOB to account for the background signal (Figure 3b).

### 2.4 Inhibition of the aggregation of Aβ42

**FIGURE 4:**
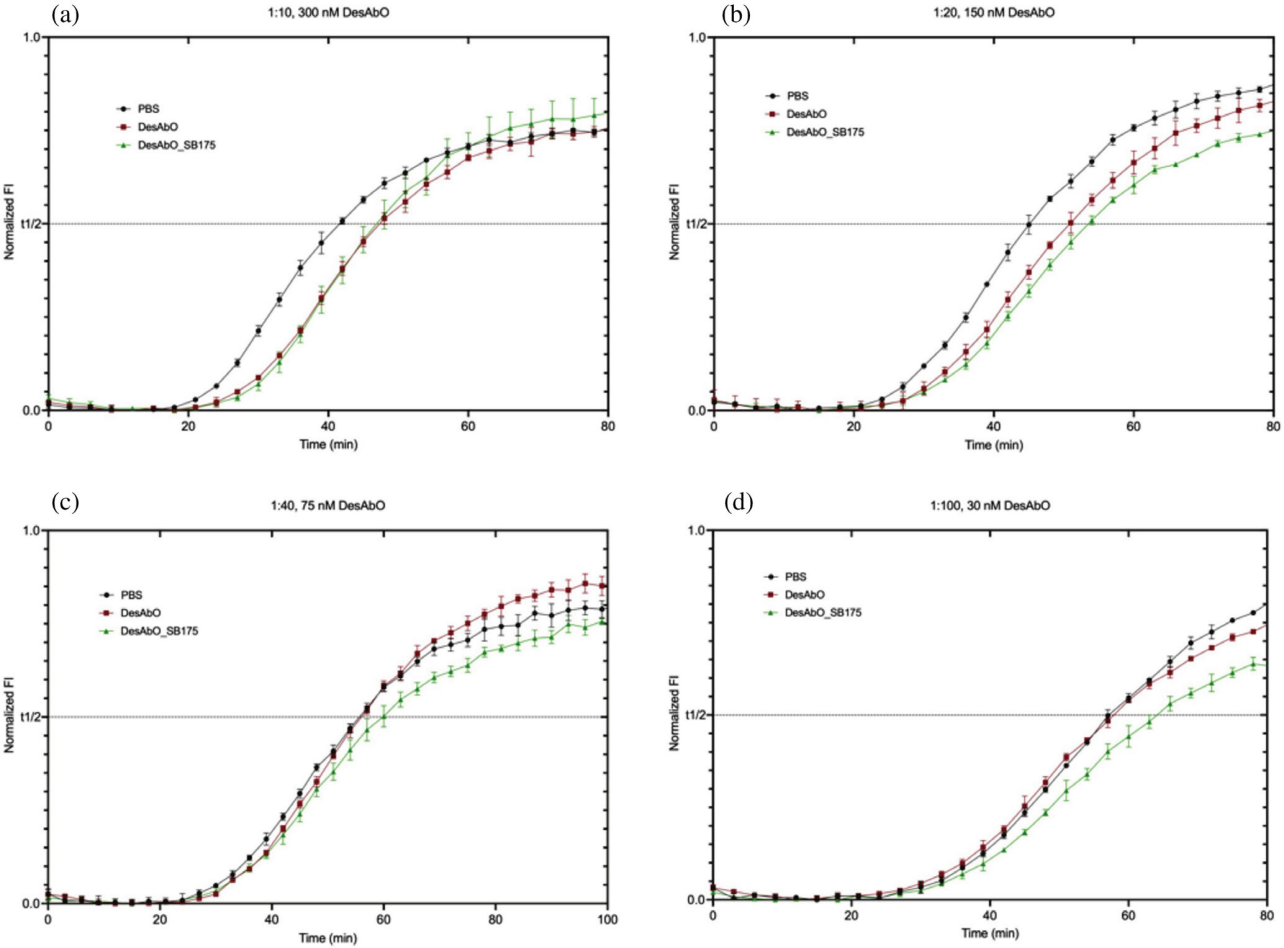
Inhibition of in vitro Aβ42 aggregation by SB175‐DesAbO. (a–d) Thioflavin T fluorescence curves of Aβ42 with varying concentrations of DesAbO and SB175‐DesAbO. The Aβ42 concentration was constant (3 μM) while the SB175‐DesAbO concentration was progressively decreased: (a) 300 nM, (b) 150 nM, (c) 75 nM and (d) 30 nM. Each DesAbO concentration was matched with a phosphate‐buffered saline (PBS) control containing the same added volume of PBS. Consequently, the ratio of PBS to sodium phosphate varied across panels (a–d), which explains the observed differences in aggregation kinetics between controls.

**FIGURE 5:**
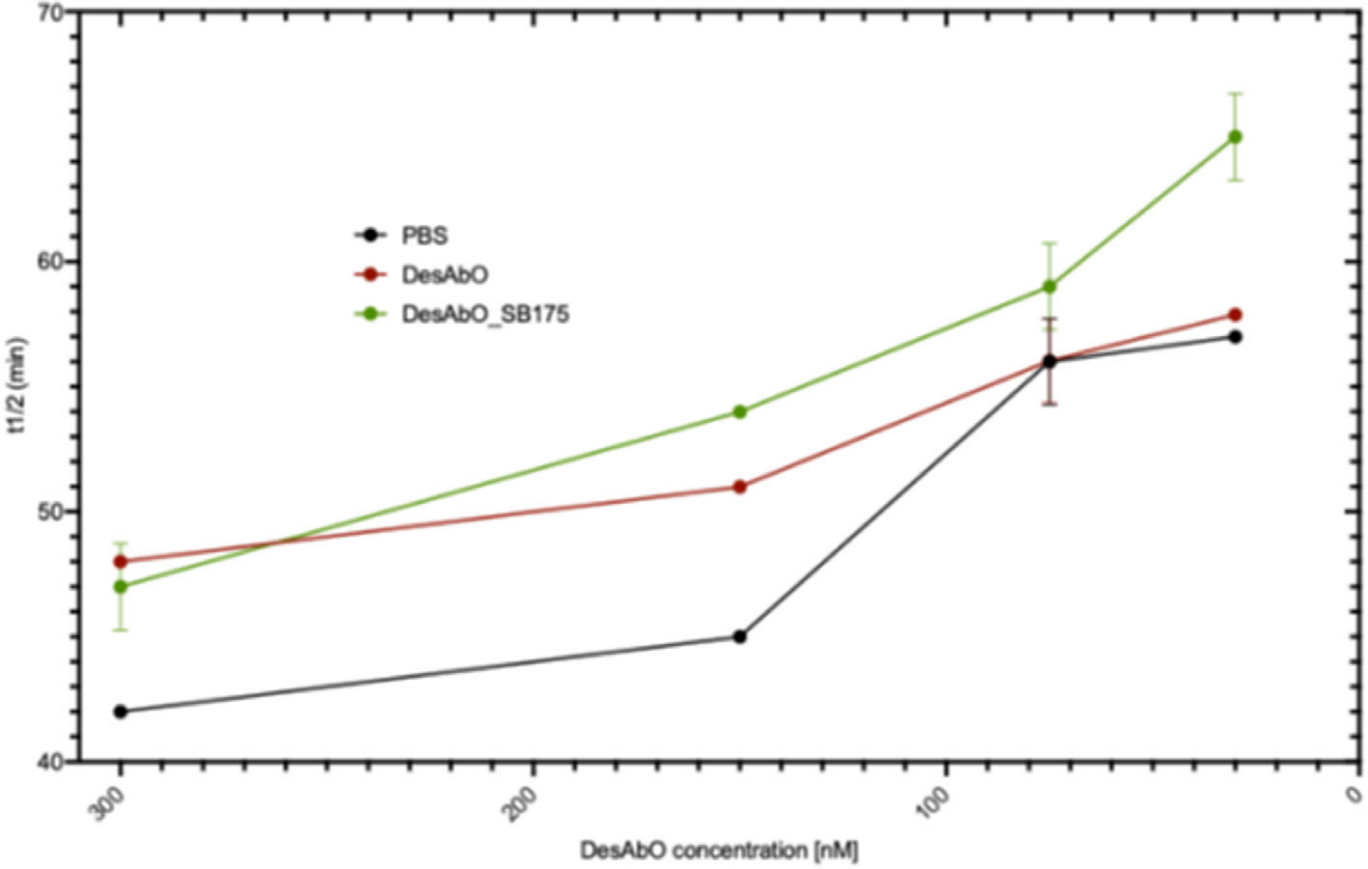
Half‐time of in vitro Aβ42 aggregation as a function of DesAbO concentration. Each nanobody concentration is shown together with its matched phosphate‐buffered saline (PBS) control, which received the same added volume of PBS. Because the proportion of PBS varied across the titration, the control half‐times vary systematically with nanobody concentration; this reflects the change in buffer composition and not an effect of the nanobody. Each condition should therefore be compared with its own control, and absolute half‐times are not directly comparable across the titration. Relative to their matched controls, SB175‐DesAbO delayed aggregation across the whole titration, whereas monomeric DesAbO did so at 300 and 150 nM but had little effect at 75 and 30 nM.

To test how DesAbO multimers inhibit aggregation of Aβ42 when compared to DesAbO, we performed aggregation experiments where different concentrations of DesAbO were added to Aβ42 and the aggregation curves were measured using thioflavin T (ThT) fluorescence. We compared DesAbO with the best‐performing DesAbO multimer, the SB175 trimer. At higher concentrations of DesAbO (300 nM), both DesAbO and SB175‐DesAbO delayed aggregation equally. The half‐time of aggregation (*t*_1/2_, horizontal dotted line) was the same for both of them (Figures 4 and 5). As the concentration was decreased to 75 and 30 nM, monomeric DesAbO had little effect on the aggregation kinetics relative to its matched control, whereas SB175‐DesAbO continued to delay aggregation (Figures 4 and 5). Concentrations are reported per construct rather than per binding domain, so 30 nM of the trimer corresponds to a nominal paratope concentration of ~90 nM. Comparing the constructs in terms of the delay relative to their matched controls, 30 nM trimer (~90 nM paratopes) gave Δ*t*_1/2_ = 8 min, whereas monomeric DesAbO gave 0 min at 75 nM and 6 min at 150 nM. The monomer, therefore, produced a smaller delay than the trimer even when supplying 150 nM of paratopes, well above the 90 nM of trimers. Were the three paratopes of the trimer acting independently, 30 nM trimer and 90 nM monomer would be equivalent. This is consistent with an additional contribution from multivalent engagement, although we have not measured the number of accessible paratopes per construct, which may be fewer than the nominal valency.

### 2.5 AlphaFold modeling of the DesAbO multimer structures

**FIGURE 6:**
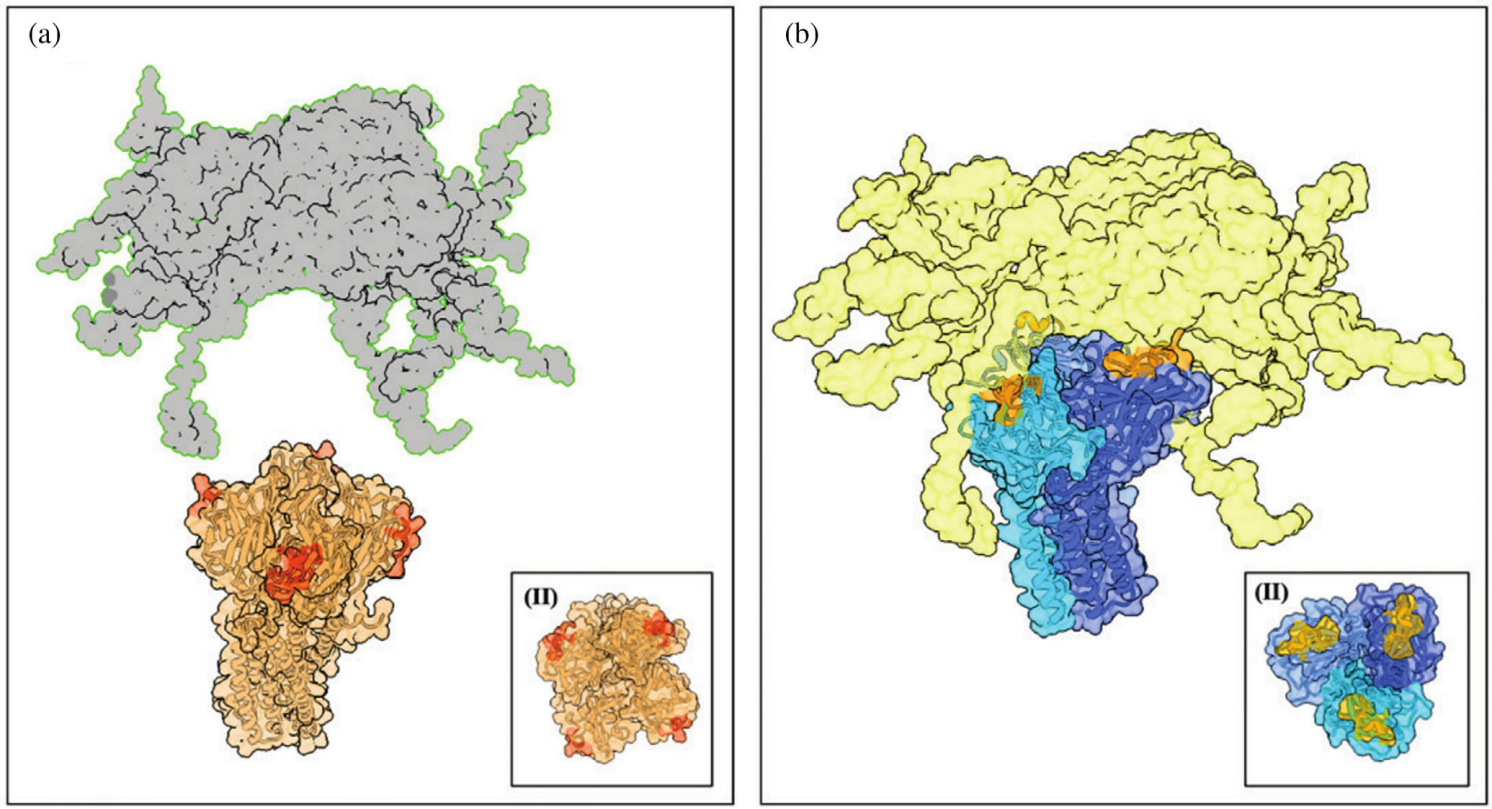
Illustrative AlphaFold models of two DesAbO multimers. (a) DesAbO tetramer. The multimerization domain, which forms the scaffold holding the nanobodies together, is shown in light orange. It comprises four identical, non‐covalently associated units, each carrying a covalently bound DesAbO nanobody (dark orange). The region outlined in green denotes an Aβ42 oligomer. (I) Side view; (II) top view. (b) DesAbO SB175 trimer. The multimerization domain is shown in shades of blue, each shade corresponding to one unit of the trimeric domain, with the nanobodies in orange and an Aβ42 oligomer in light yellow. (I) Side view; (II) top view. In both panels the Aβ42 oligomer is included for illustration only as its structure and epitope spacing are not known at this level of detail, and the model does not represent a determined structure. The models likewise illustrate plausible arrangements for multivalent engagement rather than validated complexes. The confidence scores of the selected models are modest (Predicted Template Modeling = 0.463 for the SB175 trimer and 0.482 for the tetramer; Table S2), and AlphaFold does not readily capture linker flexibility or conformational change upon binding.

To model the structures of the DesAbO multimers, we used ColabFold (Mirdita et al., 2022) for two promising multimers, that is, the tetramer and the SB175 trimer (Figure 6). In Figure 6a, the light orange structure is the scaffold for the tetramer, that is, the multimerization domain. It consists of four identical, non‐covalently bound units. At the end of each unit, there is a covalently bound Nb (dark orange). Under the denaturing conditions of the electrospray ionization mass spectrometry (ESI‐MS) measurement, the observed mass corresponds to a single unit of the multimerization domain with one Nb, consistent with the subunits associating non‐covalently. In Figure 6b, the different shades of blue correspond to the individual units of the protein multimerization domain that holds the Nbs together, whereas the Nbs are shown in orange and the oligomer in light yellow. These models illustrate plausible arrangements by which DesAbO multimerization could enable multivalent engagement of Aβ42 oligomers and thereby increase apparent binding avidity, but the models should be considered illustrative. Furthermore, structural models of DesAbO multimers provide approximate geometry and paratope spacing. These should be interpreted as conceptual rather than precise, since AlphaFold does not capture the flexibility of the (GGS)_3_ linkers, conformational changes upon binding, or the dynamics of Aβ42 oligomers, and since the reliability of multi‐chain interface predictions is variable (Zhu et al., 2023). The confidence scores of the selected models are modest (Predicted Template Modeling [pTM] = 0.463 for the SB175 trimer and 0.482 for the tetramer, Table S2). The Aβ42 oligomer shown in Figure 6 is included for illustration only, since neither its structure nor its epitope spacing is known at this level of detail. The paratope separations quoted above should therefore be read as approximate features of these models, which motivate a geometric hypothesis, rather than as measurements demonstrating an epitope–paratope match.

## 3 CONCLUSIONS

By engineering multimeric variants of DesAbO with diverse architectures, we demonstrated enhanced avidity and specificity for Aβ oligomers compared to the monomeric form of DesAbO. These multimers showed improved performance in both binding assays and aggregation‐inhibition studies, with the SB175 trimer emerging as a particularly promising candidate due to its favorable balance of high avidity and low non‐specific binding.

The increased binding strength and aggregation delay capabilities of the DesAbO multimers position them as promising tools for two main applications. First, they hold potential as biomarkers for early Alzheimer's disease diagnosis, offering the sensitivity required to detect the low concentrations of oligomers present in biological samples such as cerebrospinal fluid. Second, their ability to mitigate Aβ aggregation at lower concentrations highlights their therapeutic potential in preventing or slowing neurotoxicity associated with oligomer accumulation.

Despite these advances, challenges remain. Multimerization increased binding not only to Aβ42 oligomers but also to Aβ42 fibrils. We attribute this to avidity and surface rebinding rather than to a change in the intrinsic preference of the paratope, since fibrils display the same epitope repetitively along their surface, a geometry well suited to multivalent engagement. It does not amount to generic recognition of β‐sheet structure, as binding to α‐synuclein fibrils remained minimal (Figure 3), and the constructs differ from one another in their oligomer‐to‐fibril ratio (Figure 2b), which a generic β‐sheet binder would not do. The constructs are therefore best compared by this ratio rather than by their absolute binding signals, and further optimization is required to balance avidity against selectivity. One route would be to select a multimerization geometry matched to the epitope spacing of oligomers while mismatched to the fibril repeat.

A further open question is whether these constructs discriminate between oligomers formed early and late in the aggregation reaction. The oligomers used here represent a single preparation, and resolving this would require time‐resolved sampling of the aggregation process with independent characterization of the species present at each time point. The answer is likely to matter differently for the two applications considered here, since a diagnostic reagent may need to detect the earliest species present, whereas a therapeutic agent may benefit from engaging the later species that participate in secondary nucleation.

The aggregation experiments reported here were performed at a single Aβ42 concentration and are therefore not suited to identifying which microscopic step of aggregation is inhibited. Since the multimers bind both oligomers and fibrils, two contributions are possible and not mutually exclusive: binding to the fibril surface could block the sites at which secondary nucleation occurs, thereby suppressing the dominant source of new aggregates under these conditions (Cohen et al., 2013), and binding to oligomers could sequester them and slow their conversion into fibrils. Distinguishing between these, and establishing whether either predominates, would require a global kinetic analysis of aggregation curves acquired across a range of monomer concentrations, together with seeded assays in which elongation dominates (Linse et al., 2020). We regard this as an important direction for future work.

Overall, our findings establish multimerized DesAbO Nbs as versatile and potent tools for investigating and potentially addressing the role of Aβ oligomers in Alzheimer's disease pathology. More broadly, our results indicate that the geometry of multimerization, and not valency alone, determines conformational selectivity, a principle likely to apply to the targeting of oligomeric species in other protein misfolding diseases.

## 4 MATERIALS AND METHODS

### 4.1 Design of the multimers

All the multimers were designed using the following template, starting from the N‐terminus of the Nb: Nb–(GGS)_3_–multimerization domain–(GGS)_1_–6× His, where the Nb is DesAbO, (GGS)_3_ is a triple glycine–glycine‐serine linker between the Nb and the multimerization domain, and 6× His is the His tag for the purification and ELISA (Figure 1). The multimerization domain determines the geometry and multimer number of the Nb (the trimer or tetramer, e.g.). The amino acid sequence of DesAbO is the following, with the paratope underlined (Aprile et al., 2020):

MEVQLVESGGGLVQPGGSLRLSCAASGFNIKDTYIGWVRRAPGKGEEWVASIYPTNGYTRYADSVKGRFTISADTSKNTAYLQMNSLRAEDTAVYYCAAGSESAFGRAEEEAAWGQGTLVTVSSGSHHH. The amino acid sequences of the multimerization domains are reported in Table S1.

### 4.2 Expression, purification, and characterization of the DesAbO multimers

DesAbO and its multimeric variants were expressed in pET28a(+) *Escherichia coli* vectors. After ensuring the proper placement of the restriction enzymes, T7 promoter, LacO, and kanamycin resistance within the GenScript sequence, multimeric variants ranging from trimers to octamers were ordered. Plasmids encoding DesAbO wild‐type (WT), DesAbO T4 fibritin (trimeric variant), DesAbO SB175 (trimeric variant), DesAbO 2L8HC4_12 (tetrameric variant), DesAbO HALC5–15_262 (pentameric variant), DesAbO HALC6–18_265 (hexameric variant), and DesAbO WSHC8 (octameric variant) were cloned and then used for protein expression in SHuffle T7 *lysY* competent *E. coli* cells, following the manufacturer's protocol. The cell cultures were pre‐cultured with kanamycin and lysogeny broth media, and the pre‐culture was used to grow the scaled‐up culture. The bacteria were induced at the mid‐log growth stage (0.6–0.8 optical density) with isopropyl β‐D‐1‐thiogalactopyranoside, and the cytoplasmic cell content was subsequently lysed using tip sonication (2 s ON, 4 s OFF, 40% amplitude, 5 min sonication time).

Purification was performed via Nickel‐IMAC, with 40 and 200 mM imidazole in 1× PBS being the eluting buffers. Finally, the samples were dialyzed against 1× PBS and further purified by size exclusion chromatography (SEC; Superdex Increase 200, 10/300 GL, Cytiva) using 1× PBS. The concentration was measured using NanoDrop (Thermo Scientific) at 280 nm wavelength and extinction coefficients computed using the amino acid sequence (Benchling). For the trimeric variants, the extinction coefficient was assumed to be triple that of the monomers; for tetramers, quadruple, etc.

The soluble proteins were then analyzed using ESI‐MS (Figure S1), which indicated the correct masses of the formed multimers. ESI‐MS was performed under reducing conditions, and as the interactions between the monomeric units of the multimer are non‐covalent, the expected observed mass is the sum of one unit of the multimerization domain and one Nb.

Multimers were also shown to be correctly folded via CD (Figure S2A) and Tycho (Figure S2B) with the fusion to oligomeric protein domains not significantly altering the core Nb structure of DesAbO. Following Tycho profiling, the melting temperature of the T4 trimeric variant was found to be the highest, with the pentamer following closely thereafter. All multimers, with the exception of the tetramer, displayed comparable melting temperatures to DesAbO, indicating that the addition of these domains did not negatively alter the stability and structure of DesAbO.

Following expression, the hexameric variant yielded consistently high impurity rates, so longer SEC fraction collection times were implemented to separate out any unwanted contaminants. Nb concentrations were calculated by assuming multimeric values, for example, the trimer was assumed to have an extinction coefficient for the Beer–Lambert law three times that of its denatured sequence. Following all characterization, remaining samples were flash‐frozen in liquid nitrogen and stored at −80°C.

### 4.3 Size exclusion chromatography of DesAbO multimers

Superdex 75 Increase 10/300GL and Superdex 200 Increase 10/300GL columns were equilibrated with pH 7.4 1× PBS on a Cytiva Akta Pure. WT DesAbO was purified on the Superdex 75 column, while DesAbO multimers were purified on the Superdex 200 column. After pre‐equilibration, 1–2 mL portions of concentrated DesAbO solutions were injected onto the column, and the protein was eluted using pH 7.4 1× PBS with an 0.8 mL/min flow rate (Superdex 75) or an 0.75 mL/min flow rate (Superdex 200). The protein‐containing fractions were then collected and analyzed using SDS‐PAGE and ESI‐MS. Purified DesAbO was then flash‐frozen using liquid nitrogen and stored at −80°C for later use. All concentrations are reported as the molar concentration of the Nb construct (e.g., monomer and trimer), without adjustment for the number of binding domains per construct.

### 4.4 Expression, purification, and characterization of Aβ42

Aβ42 was produced as described previously (Linse, 2020). After production and purification, Aβ42 aliquots were flash‐frozen in liquid nitrogen and stored at −80*°*C.

### 4.5 In vitro Αβ42 aggregation assay

Lyophilized Αβ42 was dissolved in 0.5 mL of 6M guanidine hydrochloride (GnHCl) and incubated on ice for 3 h. Αβ42 was then purified using SEC using a Superdex 75 10/300 GL column (Cytiva) equilibrated in 20 mM sodium phosphate (NaP), pH 8.0, 0.2 mM ethylenediaminetetraacetic acid (EDTA). The peak corresponding to the monomeric peptide was then collected. The concentration was calculated using the mean peak absorbance and an extinction coefficient of *ε*_280_ = 1495 M^−1^ cm^−1^. Purified Aβ42 at a final concentration of 5 μM in 20 mM NaP, pH 8.0, 0.2 mM EDTA was mixed with 50 μM ThT. One hundred microliters per well of the Αβ42/ThT mix was added to three wells of a 3881 low‐binding plate (Corning, 96 wells). The aggregation was carried out under quiescent conditions at 37°C in a Clariostar plate reader (BMG Labtech). The ThT fluorescence was tracked until it reached the plateau (fluorescence emission at 480 nm and excitation at 440 nm).

### 4.6 Preparation of Αβ42 oligomers and fibrils

In vitro Aβ42 oligomers and fibrils were generated using a modified version of the aggregation assay described above. Aβ42 was diluted to 5 μM in aggregation buffer, and the half‐time of aggregation (*t*_1/2_), defined as the time at which 50% of monomers are converted into fibrils, was determined from ThT fluorescence measurements (Figure S3). A separate aggregation assay was then set up using the same conditions, with three wells containing Aβ42 and ThT to monitor aggregation kinetics, and additional wells containing Aβ42 without ThT to generate material for ELISA, as ThT may interfere with the assay signal. At the end of the aggregation reaction, the samples intended for oligomer analysis were centrifuged at 15,000 rpm for 15 min at room temperature to remove fibrillar species, and the supernatant, an oligomer‐enriched soluble fraction, was collected. This preparation follows the protocol of (Aprile et al., 2020) in which Aβ42 oligomers obtained in the same way were characterized by atomic force microscopy morphology mapping and super‐resolution direct stochastic optical reconstruction microscopy imaging. The size distribution of the preparations used in the present study was not determined independently; the fraction is used here as a common antigen for the comparison of the DesAbO constructs rather than as a defined molecular species. Direct structural characterization of these preparations would place this attribution on a firmer footing and is an important step for future work.

### 4.7 Enzyme‐linked immunosorbent assay

For the purposes of this study, we adapted a previously reported ELISA protocol (Aprile et al., 2020), using DesAbO multimers as primary antibodies and Αβ42 oligomers, fibrils, and monomers as the bound antigens. The oligomers (2.5 μM) and fibrils (2.5 μM), prepared as described above, as well as monomers of Αβ42 (2.5 μM), and NaP buffer (20 mM NaP, pH 8, 0.2 mM EDTA) were plated on a 96‐well Maxisorp (NUNC) plate, 70 μL/well, triplicates, and incubated for 1 h at room temperature with no shaking, sealed to prevent evaporation. The plate was then washed 3 times with PBS‐T (0.05%), 300 μL/well. The plate was then blocked with 5% bovine serum albumin (BSA) (Fluka) in PBS‐T (0.05%) and incubated overnight at 4°C, shaking at 250 rpm, sealed. The following day the plate was washed six times with phosphate‐buffered saline with Tween (PBS‐T) (0.05%), 300 μL/well. Next, 5 μM DesAbOs were incubated for 1 h, 250 rpm, room temperature, 70 μL/well, sealed. The plate was again washed three times with PBS‐T (0.05%), 300 μL/well. The secondary antibody, anti‐His‐HRP (Abcam, 1 mg/mL stock) was diluted 1:5000 in 5% BSA in PBS‐T (0.05%) and added to the wells (70 μL/well). It was incubated for 40 min, room temperature, 250 rpm, sealed, and then washed three times with PBS‐T (0.05%), 300 μL/well. Next, 100 μL/well of 3,3′,5,5′‐tetramethylbenzidine (Thermo Scientific) was added and the plate was allowed to develop for 10 min in the dark, after which 2M HCl was added to each well, with 50 μL/well. The absorbance at 450 nm was then read using the Clariostar (BMG Labtech).

### 4.8 Aggregation inhibition

The aggregation inhibition was performed as described above. For each DesAbO concentration tested, various volumes of DesAbO stock (prepared in PBS) were added to monomeric Αβ42 (3 μM) samples with 50 μM ThT before loading into the aggregation plate. The corresponding negative control contained the same volume of PBS instead of DesAbO. As a result, the proportion of PBS varied across different conditions, leading to minor differences in the aggregation kinetics of Aβ42 controls shown in Figure 4. The final concentrations of DesAbO were 300, 150, 75, and 30 nM.

### 4.9 AlphaFold predictions

The multimer structure predictions were done using ColabFold (Mirdita et al., 2022), which combines AlphaFold2 (Evans et al., 2021; Jumper et al., 2021) with MMSeqs2 (Steinegger & Söding, 2017). The following parameters were selected: *multiple sequence alignment (MSA) Options*. MSA Mode: mmseqs2_uniref_env. Pair mode: unpaired_paired. *Advanced Settings*. Model_type: auto. if auto selected, will use num_recycles = 20 if model_type = alphafold2_multimer_v3. recycle_early_stop_tolerance: auto. For both the trimer and tetramer, five multimer structures were computed and ranked based on the pTM score. The pTM measures the agreement between two folded proteins and estimates the quality of the complex; although the reliability of AlphaFold‐Multimer predictions for multi‐chain complexes varies with the interface (Zhu et al., 2023). The chosen structure in this paper is the top‐ranked structure. The ranked structures have the pTM scores reported in Table S2.

## AUTHOR CONTRIBUTIONS

**Magdalena Nowinska:** Conceptualization; methodology; software; data curation; investigation; validation; formal analysis; writing – original draft; writing – review and editing. **Casey Mogilevsky:** Conceptualization; methodology; investigation; validation; formal analysis; writing – review and editing. **Michele Vendruscolo:** Conceptualization; methodology; data curation; formal analysis; supervision; funding acquisition; visualization; project administration; resources; writing – original draft; writing – review and editing. **Elijah Suh:** Conceptualization; methodology; data curation; investigation; validation; formal analysis; writing – original draft; writing – review and editing.

## Supporting information


**Data S1.** Supporting Information.

## Data Availability

The data that support the findings of this study are available on request from the corresponding author. The data are not publicly available due to privacy or ethical restrictions.
