# Supplementary material for: Multimerization of a rationally designed nanobody for enhanced avidity toward Aβ42 oligomers
Source: Protein Sci. 2026 Aug 19;35(9):e70754. doi: 10.1002/pro.70754 (PMC13488367; doi:10.1002/pro.70754)
Supplement: Supplementary file 1 [file PRO-35-e70754-s001.docx]

**SUPPLEMENTARY INFORMATION**

**Multimerization of a Rationally Designed Nanobody for Enhanced Avidity towards Aβ42 Oligomers**

Magdalena Nowinska^‡^, Elijah Suh^‡^, Casey Mogilevsky and Michele Vendruscolo^*^

*Centre for Misfolding Diseases, Yusuf Hamied Department of Chemistry,*

*University of Cambridge, Cambridge CB2 1EW, UK*

**Table S1. Amino acid sequences of the multimerization domains used in this work.**

| **Multimerization** **domain** | **Amino acid sequence** |
| --- | --- |
| Trimer (SB175)  (Hunt et al., 2022) | SEALEELEKALRELKKSTDELERSTEELEKNPSEDALVENNRLIVENNKIIVEVLRIIAKVLK |
| Trimer (T4 Fibritin)  (Güthe et al., 2004) | GYIPEAPRDGQAYVRKDGEWVLLSTFL |
| Tetramer (2L8HC4_12)  (Boyken et al., 2016) | TAIEANSRMLKALIEIAKAIWKALWANSLLLEATSRGDTERMRQWAEEAREIYKEAKKIIDEADEIVKEAKERHD |
| Pentamer (HALC5–15_262)  (Wicky et al., 2022) | DVPLTDPKNLNEFLYALGEGLKGMKNLKKLTLTFPSNPLTIPGDISEGFRELGEGLKGMKNLEELTVTFNDVPLTDPKNLNEFLYALGEGLKGMKNLKKLTLTFPSNPLTIPGDISEGFRELGEGLKGMKNLEELTVTFNDVPLTDPKNLNEFLYALGEGLKGMKNLKKLTLTFPSNPLTIPGDISEGFRELGEGLKGMKNLEELTVTFN |
| Hexamer (HALC6–18_265)  (Wicky et al., 2022) | NIKIPNPKDLSELLKKLGEGLKGLPNLKTLTLTLSNIELPEDADLSPGAEGLGEGLKGLPNLETLTFTISNIKIPNPKDLSELLKKLGEGLKGLPNLKTLTLTLSNIELPEDADLSPGAEGLGEGLKGLPNLETLTFTISNIKIPNPKDLSELLKKLGEGLKGLPNLKTLTLTLSNIELPEDADLSPGAEGLGEGLKGLPNLETLTFTIS |
| Octamer (WSHC8)  (Xu et al., 2020) | SAEELLRRSREYLKKVKEEQERKAKEFQELLKELSERSEELIRELEEKGAASEAELARMKQQHMTAYLEAQLTAWEIESKSKIALLELQQNQLNLELRHI |

**Table S2.** **AlphaFold predicted structures rank parameters (trimer and tetramer).**

|  | **SB175 trimer** | | **Tetramer** | |
| --- | --- | --- | --- | --- |
| **Rank** | **Number of recycles** | **pTM** | **Number of recycles** | **pTM** |
| 1 | 8 | 0.463 | 11 | 0.482 |
| 2 | 6 | 0.446 | 6 | 0.457 |
| 3 | 7 | 0.442 | 20 | 0.415 |
| 4 | 20 | 0.427 | 13 | 0.356 |
| 5 | 20 | 0.409 | 20 | 0.333 |

**Figure S1.** **Deconvoluted ESI-MS spectra for the DesAbO multimers.** Deconvoluted ESI-MS spectra of DesAbO and its multimeric variants. Spectra are shown for DesAbO (WT) and for each multimerisation construct: the T4 trimer, the SB175 trimer, the tetramer, the pentamer, the hexamer and the octamer. In each case the major deconvoluted peak corresponds to the mass expected for a single multimerisation-domain subunit covalently linked to one DesAbO nanobody, confirming both the identity of the constructs and the conditions under which the multimers dissociate into their constituent units.

**Figure S2. Characterization of the thermodynamic stability of DesAbO multimers. (A)** Far-UV CD spectra of DesAbO (WT) and the multimeric variants at 20 °C. The spectra of the SB175, tetrameric, pentameric, hexameric and octameric variants are dominated by the α-helical signature of their multimerisation domains, with minima near 208 and 222 nm; the T4 trimer instead carries a fibritin domain and its spectrum differs accordingly. The β-sheet contribution of the nanobody is not resolved separately from the helical signal. **(B)** Tycho thermal denaturation curves for DesAbO (WT) and the computationally designed multimeric variants, reported as the ratio of intrinsic fluorescence at 350 and 330 nm. **(C)** The corresponding curve for the T4 trimer, shown separately because its 350/330 nm ratio lies on a different scale. The tetramer showed no clear unfolding transition. This does not indicate hyperstability: it is equally consistent with a broad or non-cooperative transition, with partial disorder before the temperature ramp, or with aggregation on heating, and the Tycho measurement alone cannot distinguish among these. The CD spectrum in panel (A) argues against the tetramer being substantially disordered at the starting temperature, although it does not report on its behaviour at elevated temperature. The lower conformational selectivity of the tetramer for Aβ42 oligomers (**Figure 2B**) is not attributed to misfolding, since these data do not establish a connection between the Tycho profile and the binding behaviour.

**Figure S3.** **Normalized kinetics of Αβ42 aggregation using ThT fluorescence.** The half-time (t_1/2_) of aggregation is the time point at which 50% of the monomer conversion into fibrils has taken place. The concentration of Αβ42 was 5 μM. The half-time is at 117 mins and is marked with a green symbol.

**Supplementary References**

Boyken SE, Chen Z, Groves B, Langan RA, Oberdorfer G, Ford A et al. (2016) De novo design of protein homo-oligomers with modular hydrogen-bond network–mediated specificity. *Science* 352:680-687.

Güthe S, Kapinos L, Möglich A, Meier S, Grzesiek S, Kiefhaber T (2004) Very fast folding and association of a trimerization domain from bacteriophage T4 fibritin. *J Mol Biol* 337:905-915.

Hunt AC, Case JB, Park Y-J, Cao L, Wu K, Walls AC et al. (2022) Multivalent designed proteins neutralize SARS-CoV-2 variants of concern and confer protection against infection in mice. *Sci Transl Med* 14:eabn1252.

Wicky B, Milles L, Courbet A, Ragotte R, Dauparas J, Kinfu E et al. (2022) Hallucinating symmetric protein assemblies. *Science* 378:56-61.

Xu C, Lu P, Gamal El-Din TM, Pei XY, Johnson MC, Uyeda A et al. (2020) Computational design of transmembrane pores. *Nature* 585:129-134.
